# Evaluation of a hybrid antimicrobial restriction process at a large academic medical center

**DOI:** 10.1017/ash.2021.188

**Published:** 2021-10-21

**Authors:** Jesse D. Smith, Linh H. Nguyen, Tamara Krekel, Jerrica Waggoner, David J. Ritchie, Michael J. Durkin, Kevin Hsueh, Elizabeth A. Neuner

**Affiliations:** 1 University of Health Sciences and Pharmacy in St. Louis, St. Louis, Missouri; 2 Department of Pharmacy, Barnes-Jewish Hospital, St. Louis, Missouri; 3 Center for Clinical Excellence, BJC Healthcare, St. Louis, Missouri; 4 Pharmacy Practice Department, University of Health Sciences and Pharmacy in St. Louis, St. Louis, Missouri; 5 Division of Infectious Diseases, Washington University School of Medicine, St. Louis, Missouri

## Abstract

We conducted a retrospective review of a hybrid antimicrobial restriction process demonstrating adherence to appropriate use criteria in 72% of provisional-only orders, in 100% of provisional orders followed by ID orders, and in 97% of ID-initiated orders. Therapy interruptions occurred in 24% of provisional orders followed by ID orders.

Preauthorization is a foundational intervention for antimicrobial stewardship programs. Preauthorization improves initial adherence to guidelines, decreases *Clostridioides difficile* infections (CDIs), and reduces antimicrobial use and costs.^1,2^ However, a strict preauthorization policy places a heavy workload on the authorizing providers (particularly after hours), contributes to a perceived loss of prescriber autonomy, and may contribute to antibiotic administration delays.^3,4^

In September 2019, the Barnes-Jewish Hospital Antimicrobial Stewardship Program (BJH-ASP) transitioned from strict preauthorization to a hybrid restriction system to improve patient safety and reduce the risk of missed orders and delays in therapy. For select restricted antimicrobials, a provisional order set was created that allowed up to a 24-hour supply to be utilized without any preauthorization. Additionally, the order set contained an optional order for an infectious diseases (ID) consultation if therapy beyond 24 hours was desired. Orders for restricted antimicrobials with a duration longer than 24 hours were limited to ID prescribers only and a separate order was required for provisional orders to continue beyond 24 hours. All provisional orders are reviewed by the ASP within 24 hours via real-time notifications when orders are placed. In this study, we sought to track and describe process measures related to restricted antimicrobial orders.

## Methods

Inpatients with at least 1 administration of a restricted antimicrobial order from September 19, 2019, to May 19, 2020, at our 1,158-bed teaching hospital were included. Restricted antimicrobials included intravenous acyclovir, ampicillin/sulbactam, ceftazidime/avibactam, ceftolozane/tazobactam, fidaxomicin, and meropenem/vaborbactam. Both acyclovir (September 19, 2019, through November 18, 2019) and ampicillin/sulbactam (September 19, 2019, through January 13, 2020) were added to the order sets only during times of shortage. Patients were excluded if the order was not administered, was a dose adjustment, or was a duplicate.

The primary outcome of the study was the appropriateness of restricted antimicrobial use, as determined by institutional guidelines (Table 1) for each restriction category (provisional only, provisional followed by ID orders, and ID initiated). Secondary outcomes included the incidence of therapy interruptions related to the antimicrobial restriction process. An interruption was defined delayed administration of the first nonprovisional dose of a restricted antimicrobial of at least 90 minutes.^5^

**Table 1.** Appropriate Use Criteria for Restricted Antimicrobials

Antimicrobial	Institution Appropriate Use Criteria^a^
Acyclovir, IV^b^	• Viral meningitis/encephalitis • Disseminated herpes simplex virus (HSV)/Varicella zoster virus (VZV) infection • HSV/VZV infection in patients unable to take enteral acyclovir
Ampicillin/sulbactam^b^	• *Acinetobacter baumannii* infections
Ceftazidime/avibactam	• Definitive treatment of infections caused by *Klebsiella pneumoniae* carbapenemase (KPC)–producing or OXA-48–producing *Enterobacterales* when other susceptible therapies are not suitable based on efficacy and/or safety concerns • Definitive treatment of infections caused by multidrug-resistant (MDR) *Pseudomonas* if the organism is susceptible to ceftazidime/avibactam and resistant to cefepime, ceftazidime, ceftolozane/tazobactam, imipenem, meropenem and piperacillin/tazobactam, when other susceptible therapies are not suitable based on efficacy and/or safety concerns.
Ceftolozane/tazobactam	• Definitive treatment of infections caused by MDR *Pseudomonas* if the organism is susceptible to ceftolozane/tazobactam and resistant to cefepime, ceftazidime, imipenem, meropenem and piperacillin/tazobactam, when other susceptible therapies are not suitable based on efficacy and/or safety concerns
Fidaxomicin	• Definitive treatment of recurrent *Clostridioides difficile* infection (CDI) • Definitive treatment of an initial CDI in patients at high risk for recurrence (age ≥65 years plus severe disease or receiving concomitant antibiotics)
Meropenem/vaborbactam	• Definitive treatment of a KPC-producing *Enterobacterales* resistant to ceftazidime/avibactam but susceptible to meropenem/vaborbactam • Alternative to ceftazidime/avibactam combination therapy, meropenem/vaborbactam can be considered for combined coverage of KPC-producing *Enterobacterales* AND meropenem/vaborbactam-susceptible gram-positive organism

a Indications outside of the appropriate use criteria will be allowed on a case-by-case basis after discussion with the Barnes-Jewish Hosptial Antimicrobial Stewardship Program.

b Appropriate use criteria during times of national shortage

The study was approved by the local institutional review boards.

## Results

During the 8-month study period, 177 orders for restricted antimicrobials were placed. Orders were excluded due to no administration (n = 22), dose adjustments (n = 4), and duplicates (n = 2). In total, 149 orders were included: 43 provisional-only orders, 68 provisional followed by ID orders in 34 patients, and 38 ID-initiated orders (Table 2). Most provisional-only orders were placed in the emergency department (28%), compared to the medical intensive care unit (38%) for the provisional followed by ID orders. The most common restricted antimicrobials ordered differed among groups: intravenous acyclovir (n = 23, 53%) for provisional-only orders and (n = 21, 62%) provisional followed by ID orders compared to ceftolozane/tazobactam (n = 18, 47%) for ID-initiated orders. Adherence to appropriate use criteria was 72% in provisional-only orders, 100% for provisional followed by ID orders, and 97% for ID-initiated orders. ID consultations were ordered for 11 (26%) of 43 provisional-only orders. Of 43 provisional-only orders, 38 (88%) had documentation of the intent for <24 hours of therapy. No provisional-only orders were repeated. A review for unintended consequences found no worsening of signs and symptoms of infection or need for subsequent antimicrobial therapy.

**Table 2.** Order Characteristics of Restricted Antimicrobials

Characteristics	Provisional Orders Only (N = 43), No. (%)	Provisional Orders Followed By ID Orders (N = 34), No. (%)	ID Initiated (N = 38) No. (%)
Patient location, ICU	18 (42)	16 (47)	10 (26)
**Department specialty at time of initial order**			
Cardiology/cardiothoracic surgery/cardiac ICU/LVAD	2 (5)	3 (9)	9 (24)
Emergency medicine	12 (28)	2 (6)	1 (3)
General internal medicine	2 (5)	4 (12)	6 (16)
General/trauma/burn surgery	10 (23)	1 (3)	2 (5)
Medical ICU	9 (21)	13 (38)	3 (8)
Neurology/neurosurgery/neuro ICU	5 (12)	4 (12)	6 (16)
Oncology/bone marrow transplant	1 (2)	6 (18)	4 (11)
Other^a^	2 (5)	1 (3)	7 (18)
**Antimicrobial**			
Acyclovir, IV	23 (53)	21 (62)	6 (21)
Ampicillin/sulbactam	13 (30)	1 (3)	5 (18)
Ceftazidime/avibactam	0	3 (9)	2 (7)
Ceftolozane/tazobactam	5 (12)	5 (15)	18 (47)
Fidaxomicin	2 (5)	1 (3)	7 (25)
Meropenem/vaborbactam	0	3 (9)	0
**Indication at time of ordering**			
Empiric	33 (76)	18 (53)	11 (29)
Definitive	5 (12)	16 (47)	26 (68)
Prophylaxis	5 (12)	0	1 (3)
**Adherent to appropriate use criteria**	31 (72)	34 (100)	37 (97)
Acyclovir, IV	19/23 (83)	21/21 (100)	6/6 (100)
Ampicillin/sulbactam	6/13 (46)	1/1 (100)	4/5 (80)
Ceftazidime/avibactam	0	3/3 (100)	2/2 (100)
Ceftolozane/tazobactam	4/5 (80)	5/5 (100)	18/18 (100)
Fidaxomicin	2/2 (100)	1/1 (100)	7/7 (100)
Meropenem/vaborbactam	0	3/3 (100)	0

Note. ID/ASP, infectious diseases/antimicrobial stewardship program; ICU, intensive care unit; LVAD, left ventricular assist device; IV, intravenous.

a Other includes 1 pulmonary and 1 otolaryngology provisional-only order; 1 colorectal surgery for provisional followed by ID order; and 1 colorectal surgery, 1 hepatobiliary surgery/transplant, 2 otolaryngology, 3 pulmonary ID-initiated–only orders.

Among the 34 provisional orders followed by ID orders, 8 patients (24%) experienced an interruption in therapy at the point of transition between the provisional and ID-initiated order: 5 patients experienced an interruption in therapy for intravenous acyclovir, 1 patient for meropenem/vaborbactam, 1 patient for ceftazidime/avibactam, and 1 patient for ceftolozane/tazobactam. The median time for the delayed administration of an ID-initiated order was 252 minutes (range, 111–650). Review of the interruptions in therapy cases identified documented reasons for 2 cases: 1 loss of intravenous access and 1 held for procedure.

## Discussion

Our restriction process, which hybridizes a provisional unrestricted system with standard preauthorization, maintained high adherence (88% overall) to institutional appropriate use criteria. Use of the restricted antimicrobials (˜14 orders per month) remained stable during the study period, and we observed no evidence of teams “gaming” the provisional unrestricted process to obtain additional doses of restricted agents. These results suggest that the hybrid process did not lead to unnecessary initiation of restricted antimicrobials. Our results are comparable to previous data suggesting that preauthorization improves appropriateness of restricted antimicrobials. Dassner et al^6^ evaluated the effect of a second-sign process for restricted antimicrobials and found appropriate use was 84.5% by general practitioners and 92.9% for ID-approved orders. These findings are comparable to the rates of adherence to appropriate use criteria in our study: 72% provisional-only orders, 100% provisional followed by ID orders, and 97% for ID-initiated orders.

Athans et al^7^ describe an approach to antimicrobial restriction based on antimicrobial stewardship goals of improving patient outcomes and safety while reducing resistance and costs. The BJH-ASP selected agents requiring preauthorization based on similar principles but also included antimicrobials affected by shortages. A survey of members of the Emerging Infections Network in 2016 reported that 70% of ID physicians were affected by antimicrobial shortages in the previous 2 years and that ASPs were highly involved in the management.^8^ Placing durations of therapy restrictions on antimicrobials experiencing shortages was a mechanism by which the ASP could monitor usage in real time. This intervention was effective for acyclovir, with 83% adherence to use criteria among provisional-only orders and 100% adherence for provisional followed by ID orders. For ampicillin/sulbactam, only 46% of provisional-only orders were adherent. This discrepancy was primarily the result of ampicillin/sulbactam use for surgical prophylaxis, not an approved indication during the shortage.

Interruptions in therapy occurred in the provisional followed by ID orders; after removal of cases with documented reasons, interruptions in therapy occurred in 18% of cases. Data suggest that delays in the first dose of effective antimicrobial therapy can cause significant patient harm; however, the impact of subsequent delays or interruptions in therapy is much less clear.^9^ Additionally, the best way to define an interruption in therapy is uncertain, although they appear to be common. Leisman et al^10^ evaluated delays from first- to second-dose antibiotics in sepsis cases using a definition of delay of ≥25% of the recommended dosing interval, and 33% of patients experienced a major delay. Even though any delay in therapy is undesirable, in this hybrid system, delays were not observed with the first dose of antimicrobial and were comparable and no worse than reports in the literature. Further work is needed to identify reasons for delays and how to prevent their occurrence. Our initial investigation did not identify trends in hours, days of the week, or primary service.

Our study has several limitations. The sample size was small, and the study was conducted at a single center. We used a retrospective design, and the study lacked a comparator arm. There is no standard definition for interruption in therapy, and while the definition in our study was based on Institute for Safe Medication Practices guidance, it was also conservative.

In conclusion, a hybrid antimicrobial restriction system demonstrated high adherence to appropriate use criteria, including short-term criteria introduced as a part of antimicrobial shortage management processes. Further work is needed to evaluate the significance of therapy interruptions and to prevent interruptions in therapy due to preauthorization processes.
